# Spontaneous Resorption of Lumbar Disc Herniation: A Narrative Review of Pathophysiology, Predictive Factors, and Clinical Decision-Making

**DOI:** 10.3390/neurosci7020030

**Published:** 2026-03-02

**Authors:** Jagoš Golubović, Bojan Jelača, Dušan Rodić, Slobodan Torbica, Srđan Stošić, Đula Đilvesi

**Affiliations:** 1 Faculty of Medicine, University of Novi Sad, Hajduk Veljkova 3, 21000 Novi Sad, Serbia; bojan.jelaca@mf.uns.ac.rs (B.J.); dusan.rodic@mf.uns.ac.rs (D.R.); slobodan.torbica@mf.uns.ac.rs (S.T.); srdjan.stosic@mf.uns.ac.rs (S.S.); djula.djilvesi@mf.uns.ac.rs (Đ.Đ.); 2 Department of Neurosurgery, University Clinical Centre of Vojvodina, Hajduk Veljkova 1, 21000 Novi Sad, Serbia; 3 Centre for Radiology, University Clinical Centre of Vojvodina, Hajduk Veljkova 1, 21000 Novi Sad, Serbia

**Keywords:** lumbar disc herniation, spontaneous resorption, intervertebral disc, macrophage, conservative management, discectomy, neurosurgery, radiculopathy

## Abstract

Lumbar intervertebral disc herniation is a common cause of low back and radicular leg pain, traditionally managed with a combination of conservative therapies and, when indicated, surgical discectomy. An intriguing phenomenon observed in many patients is the spontaneous resorption of herniated disc material over time, often correlating with significant symptom improvement. This article is presented as a narrative review synthesizing experimental, imaging, and clinical literature relevant to spontaneous disc resorption and its implications for clinical decision-making. This paper provides a comprehensive overview of spontaneous disc herniation resorption, exploring the underlying pathophysiological mechanisms and the factors that predict which herniations are likely to regress without surgery. Key mechanisms include inflammatory-mediated degradation of disc fragments, neovascularization with macrophage infiltration and phagocytosis of extruded nucleus pulposus tissue, and biological processes such as enzymatic matrix breakdown and cellular apoptosis that collectively lead to shrinkage of the herniated mass. Patient and disc characteristics that favour spontaneous resorption are identified, such as younger age, extruded or sequestered fragment type, larger initial herniation size, and robust inflammatory response on imaging, whereas certain chronic degenerative changes may reduce this likelihood. We also review current clinical guidelines and expert recommendations on when surgical intervention is warranted versus when conservative management and observation are appropriate. Understanding the probability of natural disc fragment resolution is critical in guiding treatment decisions. In the absence of severe neurological deficits or intractable pain, a period of non-operative management can often be pursued safely, given that the majority of patients experience substantial relief within a few months as discs regress. Conversely, timely surgery is advised for those with neurological compromise or refractory symptoms. By synthesizing the latest evidence on spontaneous disc herniation resorption and its predictors, this review aims to assist neurosurgeons and spine specialists in optimizing patient selection for conservative care and identifying the proper timing for surgical intervention to achieve the best clinical outcomes. Given the narrative design, conclusions are based on synthesis of heterogeneous evidence rather than formal comparative analysis.

## 1. Introduction

Lumbar disc herniation (LDH) represents a significant health concern, affecting an estimated 1–3% of the population annually, most often adults in their third to fifth decades of life. It occurs when the nucleus pulposus of an intervertebral disc extrudes or protrudes through a tear in the annulus fibrosus, potentially compressing adjacent nerve roots. This condition commonly manifests as low back pain with radiculopathy (sciatic nerve pain radiating down the leg), and in severe cases may lead to neurological deficits such as motor weakness or sensory loss. Standard treatments for LDH span a spectrum from non-operative measures—like rest, analgesic medications, physical therapy, and epidural steroid injections—to surgical intervention (typically lumbar microdiscectomy) for cases that do not improve or present with significant neurological compromise [1,2].

Conservative management is often the first-line approach because the natural history of many disc herniations is favourable. A substantial proportion of patients experience gradual symptom resolution over weeks to months with non-surgical care. Landmark clinical studies and long-term follow-ups have shown that outcomes of conservatively treated patients can be comparable to those who undergo early surgery, once sufficient time has passed. For example, improvement in pain and function occurs in a majority of patients within about 6–12 weeks of symptom onset under conservative therapy, and at one to two years after injury, overall pain and disability levels are often similar between those managed non-operatively and those treated with surgery. These findings underscore the robust inherent healing capacity in many cases of LDH [3,4].

**Figure 1:**
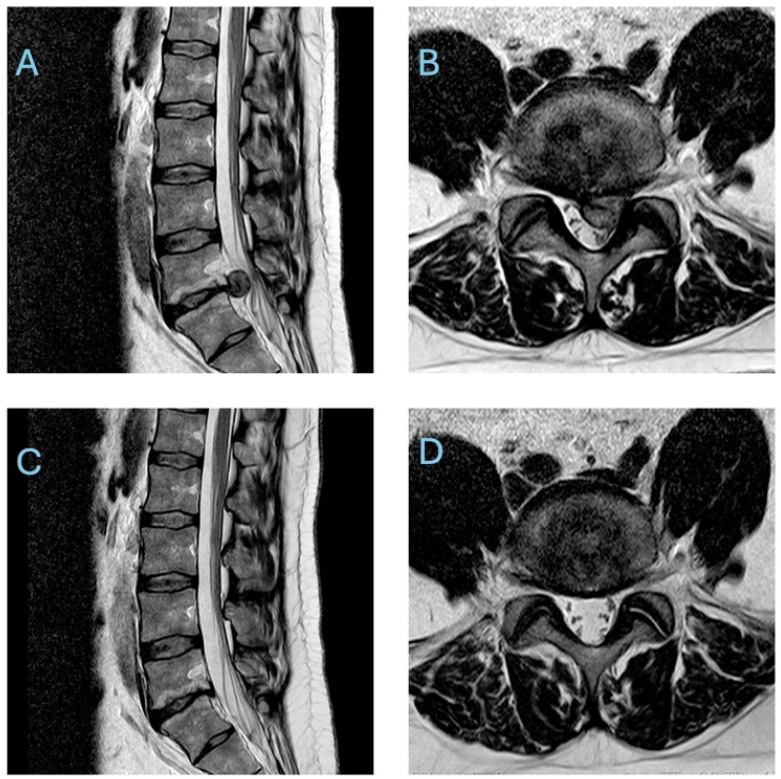
T2 weighted MRI images of a lumbar disc herniation demonstrating spontaneous resorption. The sagittal (**A**) and axial (**B**) views show a large disc extrusion compressing neural elements before treatment. After five months of conservative management, follow-up sagittal (**C**) and axial (**D**) scans of the same patient show complete disappearance of the herniated disc material, with relief of neural compression.

One of the key biological explanations for these favourable outcomes is the phenomenon of spontaneous disc herniation resorption (sometimes termed spontaneous “disappearance” or “regression” of the herniated nucleus pulposus). First noted in imaging studies several decades ago, spontaneous resorption refers to a process whereby the extruded or protruded disc fragment diminishes in size or even vanishes on follow-up imaging without any surgical removal. This correlates with the relief of nerve root compression and subsequent alleviation of radicular pain. In fact, systematic reviews have estimated that roughly half to two-thirds of herniated lumbar discs show at least partial spontaneous shrinkage on serial scans during conservative treatment, with some studies reporting complete resolution of the herniation in a significant subset of patients. It is important to note that clinical symptom improvement and radiological disc regression, while often correlated, do not necessarily occur simultaneously, and improvement in pain or function may precede, parallel, or lag behind MRI-confirmed reduction in disc size. Such evidence has provided a strong rationale for initially deferring surgery in many cases, as the body may effectively “heal” the disc extrusion over time. As illustrated in Figure 1, follow-up MRI scans of patients managed conservatively often demonstrate dramatic reduction or complete disappearance of the herniated disc fragment, corresponding to the resolution of nerve compression and symptoms [2,5,6].

Despite being well documented, the exact mechanisms driving spontaneous intervertebral disc resorption remained somewhat enigmatic for years. However, research in the past two decades has shed light on multiple physiological processes at play—including inflammation, immune cell activity, neovascularization, and tissue remodelling—that together facilitate the breakdown and absorption of herniated disc material. Concurrently, clinicians have sought to identify which patients are most likely to benefit from a non-operative course by examining predictive factors associated with disc regression (for example, the size and type of herniation on MRI, or the patient’s age and general health). Additionally, the spine care community has developed guidelines to aid in clinical decision-making: essentially, determining when it is prudent to continue conservative management versus when to escalate to surgical intervention [2,7].

This review aims to synthesize current knowledge on lumbar disc herniation and its spontaneous resorption, from both a neurosurgical and basic science perspective. We will first outline the biological and pathophysiological mechanisms thought to underlie the resorption of herniated disc tissue. Next, we will discuss the key factors that appear to predict the likelihood of spontaneous regression in a given case. Finally, we will integrate these insights with established clinical guidelines to delineate clear recommendations on patient selection for conservative management and the optimal timing of surgery when needed. The goal is to provide spine surgeons and clinicians with an evidence-informed framework to decide when to operate and when to wait in the management of lumbar disc herniation, maximizing patient outcomes while avoiding unnecessary interventions. Rather than introducing new biological mechanisms, this narrative review integrates established pathophysiological concepts with imaging predictors and guideline-based clinical decision-making. By explicitly linking biological plausibility, radiological phenotype, and timing of intervention, the review aims to support practical patient stratification—clarifying when observation is appropriate and when surgical escalation is justified in routine spine practice [1,2,4,8].

## 2. Materials and Methods

This article is a narrative review. Literature was identified through targeted searches of PubMed, Scopus, and Google Scholar, covering publications up to December 2025. Search terms included combinations of “lumbar disc herniation,” “spontaneous resorption,” “disc regression,” “macrophage,” “neovascularization,” and “conservative management”.

Eligible sources included narrative and systematic reviews, meta-analyses, clinical trials, observational imaging studies, and relevant experimental studies addressing biological mechanisms of disc resorption. Priority was given to studies reporting imaging-confirmed regression and clinically relevant outcomes. Non-English publications without accessible translations and studies unrelated to lumbar disc pathology were excluded.

Study selection and interpretation were performed by the authors based on clinical relevance and methodological rigour; no formal risk-of-bias assessment was undertaken, consistent with the narrative design.

In this narrative review, we selected studies that provided insight into the mechanisms of disc resorption (including immunological and molecular findings), epidemiological data on incidence of spontaneous regression, factors influencing outcomes, and recommendations from professional societies regarding treatment. Classic papers that first described the phenomenon of spontaneous disc regression, as well as illustrative case series and imaging studies, were also examined to contextualize the clinical relevance. Given that this is not original research involving human subjects, no institutional ethics approval was required. Our review process aimed to integrate the available evidence into practical conclusions for clinicians.

## 3. Results

### 3.1. Pathophysiological Mechanisms of Disc Herniation Resorption

Spontaneous resorption of a herniated disc is a complex biological process that involves multiple overlapping mechanisms. Broadly, the body treats the extruded disc material as a form of tissue injury or foreign substance, triggering responses that lead to gradual degradation and clearance of the disc fragment. The intervertebral disc (specifically the nucleus pulposus) is normally sequestered from the immune system by the intact annulus fibrosus and the so-called blood-disc barrier. When a disc herniates, especially if nuclear material extrudes through a torn annulus into the epidural space, this barrier is breached. The previously immune-privileged nucleus pulposus is suddenly exposed to the systemic immune surveillance. The body recognizes the extruded disc tissue as “foreign” or out-of-place, initiating an inflammatory cascade at the site of herniation [9,10].

One of the earliest responses in disc resorption is the influx of inflammatory mediators and immune cells. Herniated nucleus pulposus tissue has been shown to induce local production of pro-inflammatory cytokines such as tumour necrosis factor-alpha (TNF-α), interleukin-1 beta (IL-1β), and interleukin-6 (IL-6). These cytokines play a dual role: they contribute to pain and nerve root irritation (explaining the radiculopathy symptoms), but they also facilitate resorption by upregulating enzymes and signalling pathways that break down disc matrix. For instance, TNF-α and IL-1β stimulate intervertebral disc cells and surrounding tissues to produce matrix metalloproteinases (MMPs), which are enzymes capable of digesting proteoglycans and collagen in the disc matrix. Elevated activity of MMPs (particularly MMP-3 and MMP-7, among others) in the herniated fragment leads to degradation of the extracellular matrix that gives the disc fragment its volume, effectively “dissolving” components of the disc. In tandem, pro-inflammatory factors increase the permeability of local blood vessels and encourage neovascularization—the growth of new capillaries into the previously avascular disc tissue [10,11,12].

The formation of new blood vessels is a pivotal step in spontaneous disc absorption. Normally, the nucleus pulposus is an avascular tissue that receives nutrients by diffusion, but an extruded disc fragment that becomes vascularized allows for the ingress of circulating immune cells. Neovascularization often occurs around the periphery of the herniated fragment, which can be visualized on contrast-enhanced MRI as an enhancing rim of granulation tissue surrounding the non-enhancing disc core (sometimes called the “bull’s-eye sign”). These new capillaries deliver macrophages, the primary immune cells responsible for phagocytosis, into the disc tissue. Macrophages infiltrate the extruded material and begin to actively phagocytose (engulf and digest) the disc debris. In pathologic analyses of resorbed disc specimens, abundant macrophages are found at the site, supporting their central role in the cleanup process [11,13,14,15].

Macrophages exist in different functional states, commonly categorized as pro-inflammatory (M1) and anti-inflammatory or healing (M2) phenotypes. Both types are believed to contribute to disc resorption in a coordinated sequence. M1 macrophages, attracted by chemokines like MCP-1 released from the herniated disc, secrete further pro-inflammatory cytokines and cytotoxic substances that help break down disc tissue. M2 macrophages, on the other hand, release anti-inflammatory cytokines (such as IL-10) and growth factors that may aid in tissue repair and in resolving inflammation. The presence of macrophages not only leads directly to phagocytic removal of disc material but also sustains the production of MMPs and other enzymes through cytokine signalling, creating a positive feedback loop: inflammation recruits macrophages, which in turn amplify the degradation of the disc fragment and ultimately promote its absorption [9,11,13,14,15].

Another facet of the resorption mechanism involves the internal changes within the disc fragment. As proteoglycans are cleaved by MMPs and other proteases, the herniated nucleus pulposus loses its high water-binding capacity, resulting in dehydration and shrinkage of the fragment. This dehydration physically reduces the mass of the herniation. Concurrently, cells within the disc fragment (nucleus pulposus cells and chondrocytes) undergo stress-induced cell death pathways due to the harsh microenvironment (low nutrients, high inflammation, oxidative stress). Apoptosis (programmed cell death) of disc cells, and other forms of cell death such as autophagy and ferroptosis (an iron-dependent form of cell death linked to oxidative damage), have been implicated in disc resorption. Essentially, the herniated tissue is not only broken down from the outside by macrophages and enzymes but also “self-destructs” from the inside as its cells die and its matrix disintegrates. These processes collectively result in the fragment’s volume diminishing over time [6,11,16,17].

It is important to note that the mechanical environment plays a role as well. High mechanical stress on the spine (for example, continued heavy lifting or poor biomechanics) can aggravate inflammation and disc injury, whereas moderate, controlled motion might aid diffusion and waste removal. Some studies suggest that mechanical loading can influence disc cell metabolism—excessive stress can induce further matrix breakdown and cell apoptosis, potentially accelerating resorption, whereas gentle exercise might improve circulation to the area and promote healing [18,19].

**Figure 2:**
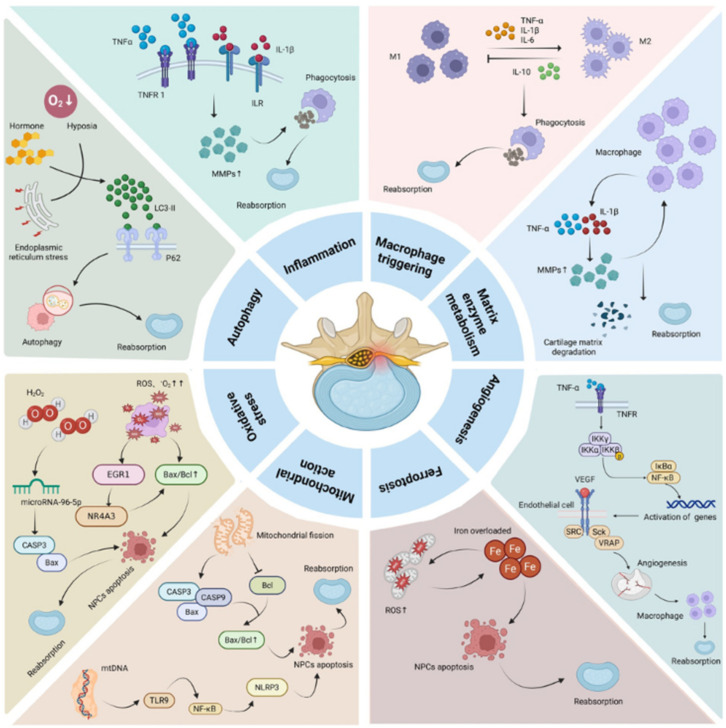
Proposed biological mechanisms of spontaneous lumbar disc herniation resorption. Extruded disc material triggers an inflammatory response: nearby cells release cytokines (e.g., TNF-α, IL-1β, IL-6) that attract immune cells and induce production of matrix-degrading enzymes (MMPs). These changes promote neovascularization, allowing macrophage cells to infiltrate the herniated fragment. Macrophages (both M1 pro-inflammatory type and M2 healing type) actively phagocytose disc tissue and secrete factors that further break down the extracellular matrix. Proteoglycan degradation leads to loss of water content (dehydration) and shrinkage of the fragment. Disc cells within the fragment undergo apoptosis or other forms of cell death under stress, adding to the tissue breakdown. Together, these processes reduce the size of the herniation and relieve nerve compression. Table 1 provides a summary of the key biological mechanisms involved in spontaneous disc herniation resorption.

The culmination of these events—matrix degradation, neovessel ingrowth, macrophage phagocytosis, inflammatory mediator activity, and cellular changes—results in the gradual shrinkage and eventual disappearance of the herniated fragment in many cases (Figure 2 and Table 1). This process typically correlates with relief of radicular symptoms, although clinical improvement may precede or occur independently of complete MRI-confirmed disc regression. Clinical imaging studies have observed that the most rapid period of resorption tends to occur in the first few months after the herniation. Significant reduction in herniation size is often seen by 3–6 months of conservative management, and in many instances the process continues such that by one year post-injury, the extruded disc has either completely resolved or dramatically reduced in size. While inflammation-mediated degradation and macrophage phagocytosis are consistently supported by human imaging and histopathological data, other mechanisms such as macrophage polarization patterns, ferroptosis, and mechanotransduction effects are derived primarily from experimental or preclinical studies and should be interpreted as emerging hypotheses rather than established clinical drivers [2,17].

### 3.2. Predictive Factors for Spontaneous Resorption

Not all herniated discs are equally likely to undergo spontaneous resorption. Clinical and radiological research has identified several factors that influence the probability and extent of disc regression. Recognizing these predictive factors can help clinicians gauge whether a patient’s herniation is a good candidate for conservative management or if it is less likely to resolve without surgery.

Herniation Type and Morphology: The nature of the disc herniation as seen on imaging is perhaps the strongest predictor of spontaneous resorption. Broadly, herniations can be classified as contained (protrusions or bulges where the disc material is still covered by some intact annulus) versus non-contained (extrusions or sequestrations where nuclear material has leaked out through the annulus). Extruded and sequestered disc fragments have a significantly higher tendency to resorb compared to contained protrusions. This is because extruded fragments are exposed to the epidural space’s immune activity; the body can more readily mount a macrophage response against a free fragment. In fact, several observational imaging studies have reported rates of spontaneous regression as high as 80–90% in selected cohorts of patients with sequestered (“free”) disc fragments, eventually demonstrating major shrinkage or complete disappearance on follow-up MRI. Extruded discs that remain connected to the disc of origin also resorb frequently, with reported regression rates in the range of approximately 60–70% in observational series. In contrast, a smaller contained protrusion (where the outer annulus is bulging but not ruptured) has a much lower chance of dramatic resorption (some studies suggest well under 50%, possibly ~30–40% or less). Broadly, one can remember that the more “out” the nucleus pulposus is, the more likely it is to be broken down and absorbed. A fully sequestered fragment is the most susceptible to resorption, followed by a broad extrusion, whereas a contained disc bulge is the least [2,10,20,21].

Size of Herniation: The initial size (volume) of the herniated disc material also correlates with resorption likelihood. Paradoxically, larger herniations—which often are extruded—tend to regress more (in absolute terms) than very small protrusions. A large disc extrusion occupying a substantial portion of the spinal canal, for example, provokes a strong inflammatory reaction, which can facilitate its removal. Moreover, larger fragments often have more extensive annular tears, allowing greater vascular penetration. On follow-up MRIs, these large herniations frequently show pronounced reduction in size. Smaller contained herniations may cause symptoms but can remain relatively unchanged if they do not elicit enough of an immune response or if they remain isolated from immune access [2,22].

Patient Age: Patient age is another factor; younger patients have been observed to experience spontaneous disc regression at higher rates than older patients. There are a few possible reasons: younger individuals generally have better overall healing capacity and a more robust immune response. Their discs also tend to have higher water content and perhaps a more vigorous vascular response when injured. In older patients, discs are more degenerated (less water content, more fibrosis) and the immune response might be less pronounced or effective in clearing the tissue. Additionally, age-related impaired circulation or comorbidities in older patients may reduce the extent of neovascularization and macrophage activity in the disc. Thus, an otherwise healthy patient in their twenties or thirties with a disc extrusion might be a prime candidate for successful spontaneous resorption, whereas a septuagenarian with a similar-looking herniation might not resorb as readily [10,17].

Sex and Hormonal Factors: Some studies have suggested that sex can play a role, although findings are not as robust as for age or herniation type. A few reports indicate that males may have a slightly higher likelihood of disc resorption than females. One hypothesis attributes this to hormonal differences—estrogen in females may modulate inflammatory and immune responses, potentially dampening the aggressive macrophage-mediated resorption to a degree. However, the difference is not stark, and both men and women can certainly experience spontaneous herniation regression. Overall health factors and the herniation characteristics likely outweigh sex as determinants, but it might be a minor consideration [10,11,12].

Body Habitus and Health: Obesity or high body weight could theoretically affect disc resorption. Patients with lower body mass index (BMI) showed higher rates of resorption in some observational studies. This might be because excess adipose tissue can produce chronic inflammatory signals that paradoxically impair a focused acute immune response, or because obesity is associated with poorer vascular health. Additionally, large body habitus places chronic mechanical stress on the spine, which could perpetuate disc irritation. On the other hand, a fit individual with good circulation might mount a more effective resorptive response. Other health factors—like smoking, diabetes, or use of certain medications (e.g., immunosuppressants or steroids)—could also influence healing. Smoking is known to impair microcirculation and could conceivably reduce neovascularization into the disc fragment, thus potentially hindering resorption. While specific data on these health factors are limited, it stands to reason that patients with fewer systemic health issues might recover more readily [23].

Degenerative Changes: The presence of chronic degenerative changes in the spine has been associated with a lower chance of spontaneous herniation shrinkage. For instance, Modic changes (degenerative edema or fibrosis in the vertebral endplates visible on MRI) often indicate an ongoing degenerative disc disease and inflammation in the area. Paradoxically, while inflammation is needed to resorb a disc, Modic changes reflect a more chronic, less acute inflammatory state and structural degeneration. Some studies have noted that herniations in segments with Modic changes are less likely to regress, possibly because the environment is not primed for the acute macrophage response seen in fresh extrusions. Similarly, a very calcified or long-standing herniated fragment might not resorb easily because it becomes essentially scar tissue over time. Timing can be a factor: a disc herniation that has been present and unchanged for years is less likely to suddenly resorb than a recent herniation, given that the initial inflammatory phase has long passed [2,10,12,17].

Among predictive factors, herniation morphology and annular rupture show the most consistent association with spontaneous resorption, whereas demographic and metabolic variables demonstrate heterogeneous and less robust evidence. Beyond these factors, each individual case can vary. But in summary, a young, healthy patient with a large, extruded (or sequestered) disc fragment is the profile most conducive to spontaneous resorption. In contrast, an older patient with a small contained protrusion and significant spinal degeneration would be less likely to see the disc resolve on its own. Table 2 outlines the major predictive factors and their influence on disc resorption outcomes.

### 3.3. Clinical Decision-Making: When to Operate vs. When to Wait

The management of lumbar disc herniation requires balancing the benefits of surgery versus the natural healing potential. Clinical guidelines generally endorse an initial trial of conservative therapy in most cases of acute LDH, with surgery reserved for specific indications or for when non-operative measures fail. The spontaneous resorption phenomenon is a major reason why a “wait-and-see” approach can be justified in appropriate patients. However, careful patient selection and timing are critical to ensure that waiting does not lead to preventable complications or unnecessary suffering. For the purposes of clinical decision-making, “progressive neurological deficit” refers to objectively worsening motor weakness attributable to nerve root compression, while “intractable pain” denotes radicular pain refractory to optimized pharmacologic and rehabilitative management and substantially impairing daily function.

Indications for Early Surgery: There are well-recognized situations where prompt surgical intervention (typically a lumbar discectomy) is strongly recommended. The clearest is cauda equina syndrome, which is a neurosurgical emergency—typically caused by a large midline disc herniation compressing the cauda equina nerve bundle. Signs include bowel or bladder dysfunction, saddle anesthesia, or severe bilateral neurological deficits. In such cases, immediate surgery is necessary to prevent permanent neurological damage. Another strong indication is the presence of progressive or significant neurological deficits in a limb, such as worsening muscle weakness or foot drop attributable to nerve root compression. If a patient shows motor deficit that is acute and worsening, most surgeons advocate for urgent removal of the compressive disc fragment rather than waiting, as prolonged compression could lead to irreversible nerve damage [24,25,26].

Beyond absolute neurological indications, intractable pain is a practical reason for earlier surgery. If a patient’s radicular pain is severe, disabling, and not adequately controlled by reasonable conservative measures (analgesics, nerve blocks, etc.), and especially if this persists for several weeks without improvement, then surgery becomes a reasonable next step even in the absence of major neurological loss. The goal in such cases is to improve quality of life and function; for instance, a patient who cannot work or sleep due to pain may choose surgery to obtain faster relief. Generally, if there has been minimal improvement in pain after around 6–8 weeks of conservative management, many guidelines suggest that surgical intervention can be offered. It’s been shown that discectomy can provide more rapid symptom relief and earlier return to normal activity compared to continued non-operative care, which is very valuable for some patients [8].

Arguments for Conservative Management: In the absence of the above urgent indications, most patients are initially managed conservatively because of the high likelihood of spontaneous improvement. Approximately 80–90% of patients with an acute lumbar disc herniation will experience at least partial, if not significant, symptom resolution over a period of weeks to a few months with non-surgical care. As discussed, more than half of herniations will shrink on their own. Therefore, for a patient with tolerable symptoms (or symptoms that show a trend of improvement), watchful waiting with continued conservative therapy is often appropriate. Conservative measures during this period can include rest (avoiding heavy lifting and acute strain), nonsteroidal anti-inflammatory drugs (NSAIDs) or other analgesics, physical therapy focusing on gentle exercise and core strengthening, and epidural steroid injections for pain relief. The patient is monitored periodically for any worsening. These principles align with recent WFNS Spine Committee recommendations emphasizing initial conservative management in neurologically stable patients [1,2,27].

If the patient’s pain is gradually improving and no new deficits develop, one can continue without surgery, sometimes for 3–6 months, since that is often how long natural resorption and healing may take. Many guidelines actually emphasize a trial of conservative treatment for at least 6 weeks (and up to 3 months) before considering elective surgery, as long as the patient is safe and reasonably comfortable. During this time, supportive care and patient education are key—patients are informed that recovery is likely and are advised on activity modifications and exercises. The knowledge that the herniation may literally be “shrinking” on its own can be encouraging and helps justify patience [10,28,29].

Evidence from Comparative Studies: Randomized trials and cohort studies have compared surgical and non-surgical management of disc herniation. The general finding is that surgery yields faster relief of leg pain and neurologic symptoms in the short term (first weeks to months), but by one to two years out, the outcomes between those who had surgery and those who did not are often similar in terms of pain, function, and work status. For example, the Spine Patient Outcomes Research Trial (SPORT) and other studies demonstrated that while patients who underwent microdiscectomy felt better quicker, those who opted for conservative management caught up in recovery by the 1-year mark on average. This suggests that surgery primarily alters the time course of recovery rather than the final outcome in many cases. Thus, if a patient can manage the initial period of pain, they have a good chance of doing well without surgery [30,31].

Nonetheless, it is crucial to individualize decisions. Some patients may not tolerate weeks of pain due to personal or occupational demands, while others may strongly prefer to avoid surgery altogether. Surgeons must also consider the patient’s psychological state—extended pain can lead to depression or anxiety, which may warrant intervention. Shared decision-making is encouraged: the clinician presents the likely natural history (including the possibility of spontaneous resorption) alongside the risks and benefits of surgery. The patient’s values and preferences are taken into account. When a decision is made to continue non-operative care, a structured follow-up plan should be in place. If at any point the patient’s condition deteriorates (e.g., new weakness or unbearable pain), the plan should shift toward surgical evaluation [32,33].

Moreover, choosing conservative management does not mean the patient cannot have surgery later. In fact, a common scenario is an initial period of waiting; if satisfactory improvement is achieved, no surgery is needed, but if not, then surgery is performed a bit later. Studies indicate that performing surgery after a delay (say 2–3 months of conservative care) is just as effective in the long run for most people as immediate surgery, provided no neurological emergency existed. The key is ensuring that the delay did not allow permanent nerve damage—which is why careful neurological monitoring is required [34,35].

Finally, it is worth noting the risks associated with each approach: Surgery carries small but real risks (infection, bleeding, nerve injury, anesthesia risks, and the possibility of recurrent disc herniation at that level in the future which might require another surgery). Conservative treatment avoids surgical risks, but the downside can be prolonged pain or temporary disability while waiting for improvement. Fortunately, with proper pain management and therapy, most patients can cope during the waiting period. Table 3 summarizes typical indications favouring surgical vs. conservative approaches [36,37].

## 4. Discussion

Our review highlights the remarkable capacity of the body to heal a lumbar disc herniation through spontaneous resorption, a process underpinned by coordinated inflammatory and cellular mechanisms. This knowledge carries important implications for clinical practice. Perhaps the most immediate is in patient counselling and shared decision-making. When patients are informed that their herniated disc can shrink or disappear on its own, they often become more receptive to conservative management and are less anxious about delaying surgery. Educating patients about the typical timeline of improvement (often within 2–3 months) and the signs of recovery can empower them to endure the initial discomfort with the confidence that non-surgical treatment is not “doing nothing,” but rather allowing natural healing to occur. On the other hand, it is equally important to set appropriate expectations: not every disc herniation will fully resorb, and some may eventually still require surgery if symptoms persist. Therefore, follow-up and re-evaluation are crucial components of conservative management strategies [2,38].

From a neurosurgical perspective, understanding the predictors of resorption can refine how we stratify patients. For example, seeing a large sequestered fragment on MRI might encourage the surgeon to recommend a trial of conservative care if safe, whereas a small contained protrusion in a patient with refractory pain might push one to proceed to surgery sooner since the odds of spontaneous resolution are lower. Also, recognizing negative predictors (like significant chronic degeneration at the level) can temper optimism about waiting and help justify an earlier surgical plan if the patient is suffering. In this way, personalized treatment plans can be formulated, leveraging the scientific insights into disc biology [10,39].

Another aspect to discuss is the potential for medical therapies aimed at enhancing disc resorption. While standard care currently categorizes treatment into conservative vs. surgical, emerging research suggests a middle ground could exist: minimally invasive interventions to promote the body’s resorption process. For instance, there has been experimental use of enzymes (like recombinant MMPs or chymopapain in the past) injected into herniated discs to chemically shrink them—essentially an attempt to mimic and accelerate natural resorption without a formal surgery. Other approaches under investigation include biological therapies that modulate macrophage activity or use anti-inflammatory agents to precisely target disc tissue. These avenues remain investigational, but they underscore how a deeper understanding of resorption mechanisms might translate into novel treatments that blur the line between “letting nature take its course” and surgical removal [11,21].

Our findings also reaffirm that the time factor is a critical consideration in LDH management. If a patient can afford to wait (medically and in terms of life impact), time often becomes an ally—enabling spontaneous regression to take place. However, the discussion should always weigh the patient’s current quality of life. There is a subset of patients whose pain is so excruciating or disabling that even a few weeks of it is untenable; for them, early surgery is a compassionate choice, not a failure of conservative care. The art of neurosurgery lies in identifying those who truly need an intervention versus those who will recover well given time. This requires not just clinical acumen but also empathy and patient engagement. It’s worth noting that spontaneous resorption mainly addresses the leg pain by relieving nerve root compression—it may not fully address back pain in the long term, as back pain can stem from underlying disc degeneration which may persist. Rehabilitation and core strengthening remain important during and after the recovery to address the overall spinal health. Recurrent disc herniation is another concern: some patients who improve and avoid initial surgery might still experience a re-herniation later (just as post-surgery patients can have recurrence). Ongoing healthy lifestyle practices and caution with heavy lifting are advisable for all patients, regardless of treatment path [2,10,40,41].

In summary, the paradigm in lumbar disc herniation management has shifted to a more conservative-first approach whenever feasible, thanks to the recognition of spontaneous resorption and generally favourable natural history. Neurosurgeons today are as much patient stewards during natural recovery as they are technical experts in the operating room. By combining knowledge of pathophysiology with individualized patient factors, we can optimize outcomes—operating when necessary and beneficial, but safely abstaining when nature’s processes are likely to suffice.

### Limitations and Strength of Evidence

This review is limited by its narrative design and reliance on heterogeneous literature, including observational imaging studies and experimental data. Quantitative estimates of resorption rates vary across studies due to differences in imaging protocols, follow-up duration, and outcome definitions. Randomized trials primarily compare surgical versus non-surgical outcomes rather than resorption itself. Consequently, conclusions emphasize trends and clinical patterns rather than precise effect sizes.

## 5. Conclusions

Lumbar intervertebral disc herniation is a condition with a remarkable capacity for self-healing in many cases. The phenomenon of spontaneous disc resorption—driven by inflammatory responses, macrophage activity, neovascularization, and matrix breakdown—underlies the improvements often seen in patients treated without surgery. Key factors such as herniation morphology (with extruded or sequestered fragments being the most amenable to natural regression), patient age, and absence of chronic degeneration can help predict which herniations are likely to resolve over time. Modern clinical management of LDH emphasizes a period of conservative treatment for the majority of patients, capitalizing on the body’s ability to alleviate nerve compression organically. Surgical intervention remains an indispensable tool for the cases that need it—particularly those with acute neurologic compromise or unrelenting pain—and it provides rapid relief and excellent outcomes in appropriately selected patients. However, knowing when to operate and when to wait is critical: unnecessary early surgery can be avoided in many instances by recognizing that natural resolution will occur, whereas delaying surgery in the wrong scenario can risk harm.

Ultimately, an evidence-based, individualized approach is paramount. By understanding the biology of disc herniation resorption and the clinical predictors of success with non-operative care, healthcare providers (especially spine surgeons) can make informed recommendations tailored to each patient. The goal is to achieve maximal recovery—whether through the body’s own mechanisms or via surgical means—while minimizing intervention when it isn’t needed. Ongoing research into the molecular pathways of disc resorption may further improve our ability to treat lumbar disc herniation in the future, potentially leading to therapies that harness these natural processes. For now, the interplay of sound clinical judgement and knowledge of spontaneous disc absorption ensures that patients receive timely, appropriate care, with surgery reserved for when it will truly make a meaningful difference.

## Figures and Tables

**Table 1:** Key Mechanisms of Spontaneous Disc Resorption.

Mechanism	Role in Spontaneous Resorption
Inflammatory cascade	Herniation exposes disc material to the immune system, provoking release of cytokines (TNF-α, IL-1β, IL-6, etc.) that initiate an inflammatory response. These cytokines contribute to pain but also stimulate downstream processes (like enzyme release and cell recruitment) that break down disc tissue [6,9].
Neovascularization	New blood vessel growth into the extruded disc fragment enables immune cell access. Capillaries form in the granulation tissue around the fragment, delivering macrophages and other cells that facilitate tissue removal [10].
Macrophage infiltration & phagocytosis	Macrophages migrate into the disc fragment and engulf (phagocytose) the extruded nucleus pulposus material. They secrete additional inflammatory mediators and proteolytic enzymes, actively digesting the disc components. This is a central mechanism of disc material clearance [13,14].
Matrix degradation (MMP enzymes)	Inflammatory signals upregulate matrix metalloproteinases and other proteases in the herniated disc and surrounding tissues. These enzymes degrade proteoglycans and collagen in the disc’s extracellular matrix, causing the fragment to lose structural integrity and mass [19].
Dehydration of fragment	As proteoglycans are destroyed, the disc fragment loses its ability to retain water. The loss of water content leads to shrinkage of the herniated material. Much of a disc’s volume is water, so dehydration significantly reduces the herniation size [12].
Disc cell death (apoptosis/autophagy)	Cells within the extruded disc undergo programmed cell death due to stress (inflammation, lack of nutrients, oxidative damage). Apoptosis and related processes (autophagy, ferroptosis) reduce the cellular components of the fragment. Together with matrix loss, this helps the tissue to diminish and be resorbed [15].

**Table 2:** Factors Influencing the Likelihood of Disc Herniation Resorption.

Predictive Factor	Influence on Spontaneous Resorption
Herniation type	Extruded or sequestered (free) disc fragments have a high likelihood of resorption because they are exposed to the immune system. Contained herniations (protrusions without annulus rupture) have a much lower tendency to regress spontaneously [20,21].
Initial herniation size	Larger herniated disc volumes (especially if extruded) provoke a stronger inflammatory response and tend to shrink more dramatically. Small disc bulges may not elicit enough response to significantly resorb [22].
Annular tear presence	A full-thickness annulus fibrosus tear (which allows nucleus material to escape) facilitates resorption (immune access). If the annulus is intact, resorption is limited. (This is closely related to herniation type above) [22].
Age	Younger patients are more likely to experience disc regression due to more robust healing and immune responses. Older patients (with less vascularity and more degeneration) show lower rates of spontaneous resorption [10,17].
Sex	Some evidence suggests males may have slightly higher resorption rates than females, potentially due to hormonal differences (estrogen in females might dampen acute inflammation), though the effect is modest [10,12].
Body weight (BMI)	Lower body weight is associated with better chances of resorption. Obesity might impede the process (poorer circulation, chronic inflammation, and greater mechanical stress on the disc) [23].
Degenerative changes	If advanced degeneration (e.g., Modic endplate changes, chronic disc disease) is present at the level, the herniation is less likely to regress. These chronic changes indicate a less acute inflammatory environment, reducing spontaneous resorption potential [2,10,12,17].
Lifestyle/health factors	Good overall health (non-smoker, no diabetes) may favour healing. Smoking and comorbid conditions could impair blood flow and immune efficiency, potentially lowering resorption likelihood [23].

**Table 3:** Indications for Surgical vs. Conservative Management of Lumbar Disc Herniation.

Recommend Surgery If:	Recommend Conservative (Wait) If:
- Cauda equina syndrome (bowel/bladder involvement) or severe progressive neuro deficit. - Significant motor weakness from nerve compression. - Severe, disabling radicular pain not improving after ~6 weeks. - No improvement despite adequate conservative treatment trial. - Patient desires faster relief and accepts surgical risks.	- No major neurological deficits (motor strength intact). - Symptoms are gradually improving under conservative care. - Short symptom duration so far (less than 6–8 weeks). - Pain is manageable with medications/therapy. - Patient prefers to avoid surgery and is comfortable waiting.

## Data Availability

No new data were created or analyzed in this study.

## Abbreviations

The following abbreviations are used in this manuscript:

LDH	Lumbar Disc Herniation
MRI	Magnetic Resonance Imaging
TNF-α	Tumour Necrosis Factor-alpha
IL-1β	Interleukin-1 beta
IL-6	Interleukin-6
MMP	Matrix Metalloproteinase
MMP-3	Matrix Metalloproteinase-3
MMP-7	Matrix Metalloproteinase-7
MCP-1	Monocyte Chemoattractant Protein-1
M1	M1 macrophage phenotype
M2	M2 macrophage phenotype
BMI	Body Mass Index
NSAIDs	Non-steroidal Anti-inflammatory Drugs
SPORT	Spine Patient Outcomes Research Trial
